# All-cause mortality risk in aged femoral intertrochanteric fracture patients

**DOI:** 10.1186/s13018-021-02874-9

**Published:** 2021-12-20

**Authors:** Xin-ping Li, Ping Zhang, Shi-wen Zhu, Ming-hui Yang, Xin-bao Wu, Xie-yuan Jiang

**Affiliations:** 1 Department of Geriatrics, Beijing Jishuitan Hospital, the 4th Medical College of Peking University, Beijing, 100035 China; 2 Department of Orthopaedics and Traumatology, Beijing Jishuitan Hospital, the 4th Medical College of Peking University, Beijing, 100035 China

**Keywords:** Osteoporosis, Femoral intertrochanteric fracture, Zoledronic acid, Mortality

## Abstract

**Introduction:**

The 1-year mortality rate after femoral intertrochanteric fracture is higher than that of femoral neck fracture, which also belongs to hip fracture (Cui et al. in Arch Osteoporos 14(1):55, 2019). With the application of the concept of co-management model of orthopedics and geriatrics, the short-term and long-term mortality of all types of hip fractures has decreased (Van Heghe et al. in Calcif Tissue Int, 2021, https://doi.org/10.1007/s00223-021-00913-5). However, the mortality of Chinese femoral intertrochanteric fracture patients under this model has not been reported in the literatures.

**Aim:**

This paper aims to study the risk factors of postoperative all-cause mortality in aged patients with femoral intertrochanteric fracture under the co-management model of orthopedics and geriatrics.

**Materials and methods:**

This is a single-center prospective cohort study based on the real world, under the co-management of orthopedics and geriatrics, 363 patients aged ≥ 65 years with femoral intertrochanteric fracture were enrolled and followed up for 2–3 years; 52 patients were lost to follow up. Age, gender, body mass index (BMI), history of comorbidities, hip Bone Mineral Density (BMD), fracture history, 25(OH)D level, hemoglobin level, anti-osteoporosis treatment were risk factors to be tested. Kaplan–Meier survival curves and multivariate Cox proportional hazards models were constructed to analyze the impact of factors on all-cause mortality.

**Results:**

(1) Most of the dead patients were older (the mean age was 83.4 years, compared with 79.8 years for surviving patients), with more complications and without anti-osteoporosis medication; gender, pre-fracture history, BMI, total hip BMD, hemoglobin, 25(OH)D had no difference between the dead and the living patients. (2) Elderly patients with Intertrochanteric fracture can benefit from the early treatment of Zoledronic Acid (within 3 days after the operation).

**Conclusion:**

Under the co-management of orthopedics and geriatrics, to Chinese patients with Femoral Intertrochanteric fracture, Doctors should pay more attention to their age and chronic disease, and give anti-osteoporosis treatment if allowed.

## Background

Due to the increased proportion of the elderly population, increasing life expectancy, and light labor lifestyle, the number of patients with osteoporosis and osteoporotic fractures had increased, and brought a high economic burden and nursing management challenges to patients, medical staff, and society [3, 4]. The number of osteoporosis-related fractures would grow to about 6 million and cost $25.4 billion annually by the year 2050 [3]. And Hip fracture is one of the main consequences of osteoporosis, with devastating results for the affected patients, including markedly increased subsequent fracture risk [5] and significant increased all-cause mortality [6]. Approximately 33% of men and 22% of women suffering a hip fracture will die within 1 year [7, 8]. To patients older than 75, Intertrochanteric fracture contributed more to the crude growth rate than femoral neck fracture [9]. And according to a systematic analysis, the pooled estimate of the 1-year mortality rate was 17.47% after femoral intertrochanteric fracture and 9.83% after femoral neck fracture between 2000 and 2018 [1]. Although hip fracture is discussed as a unified discussion, there is a significant difference in the incidence rate and mortality after operation for femoral intertrochanteric fracture and femoral neck fracture.

Now, clinicians believe that re-fracture [10] and all-cause mortality [11] can be reduced by early surgery [12], reduced bed rest, and anti-osteoporosis treatment [10, 13]. Especially in recent years, with the participation of orthogeriatrics, the proposal of the concept of rapid rehabilitation, the increase in the use of anti-osteoporosis drugs, and the improvement of medical care and patients' understanding of the disease, the in-hospital mortality and all-cause mortality of elderly hip fracture patients have been further reduced. It can also be seen from the repeated correction of the Nottingham Hip Fracture Score (NHFS) [14–16].

In this context, this paper aims to explore the risk factors of all-cause mortality in patients with femoral intertrochanteric fracture under the co-management of orthopedics and geriatrics.

## Patients and methods

### Study population

After the patient is admitted to the emergency department a pelvic X-ray is made as soon as possible; after diagnosing a hip fracture, a geriatrician is consulted before surgery for each patient with a hip fracture. All the patients over 65 years will be admitted to the geriatric trauma unit within the orthopedic trauma department, and geriatrics and orthopedics were managed together throughout the hospitalization. No surgery during weekends. Almost all patients used proximal femoral nail fixation, seldom of patients used dynamic hipscrew, and no patients used hip arthroplasty. Early mobilization after surgery with a physiotherapist was arranged on the first day from postoperative.

All patients were given osteoporosis health education and had basic calcium and vitamin D3. No patients had nonunion at the end of the follow-up.

### Inclusion and exclusion criteria

The inclusion criteria in our study were: (1) patients admitted with new femoral intertrochanteric fracture (≤ 3 weeks) and patients aged ≥ 65 years; (2) patients who received no anti-osteoporosis medication before, except calcium + vitamin D supplementations; (3) only unilateral fracture; (4) creatinine clearance rate was higher than 35 ml/min(Cockcroft-Gault formula).

Exclusion criteria were as follows: (1) pathological fractures caused by malignant tumors; (2) patients with secondary osteoporosis; (3) high-energy fractures and/or age below 65 years; (4) patients who have received other anti-osteoporosis medications except for ZOL and basement therapy, such as teriparatide acetate, Denosumab, after surgery;(5) patients with life expectancy less than 2 years, patients with tumor metastasis.

### Follow-up method

The physicians followed up the patients or the family members who lived with the patients by outpatient service and telephone in this study. Time was expressed in months; time to death was calculated from the date of surgery. The date of death or the last interview with the patients or the family members was used to determine the end of follow-up.

### Risk factors

Age, gender, body mass index, history of comorbidities (using CCI), hip BMD (DXA), fracture history, 25(OH)D level, and hemoglobin level were determined at the admission date. The date of ZOL use voluntarily was recorded; as the number of patients treated with anti-osteoporosis drugs other than ZOL is very small, only single digits, so this study does not include these patients. The secondary fractures were recorded during follow-up. Confounders were included in the final model if they changed the beta coefficient of the association > 5%.

### Statistical analysis

An estimated 294 cases would be needed to provide 90% power for a COX regression module of PASS15.0 software, assuming all-cause mortality after fracture was 10%, incident rate was 45%, and the hazard ratio of risk factors was 0.3, with a two-sidedαof 0.05 and 20% loss of follow-up rate.

A summary of the data was presented as mean ± SD, and/or percentage. Cases with missing values are deleted. For comparisons of patients’ age, BMI, and CCI between two groups, *t* tests were used. For comparisons of gender, patients with CCI ≥ 3, and pre-fracture and post-fracture numbers between two groups, *χ*^2^-tests were used.

Kaplan–Meier survival curves were constructed and stratified by gender, age, CCI, and dosing ZOL or not. The impact factors on all-cause mortality were analyzed in multivariate Cox proportional hazards models. Forward and backward stepwise models and the Akaike information criterion were used to determine the most parsimonious models and address potential biases. All statistical analyses were performed using SPSS IBM version 19.0 software. *P* < 0.05 was considered statistically significant.

The study was approved by the Beijing Jishuitan hospital Review Board, approbation number 201907-09-02 in 2019.

## Results

### General information

A total of 363 patients aged ≥ 65 years with femoral intertrochanteric fracture were enrolled in this prospective cohort study, the patients were consecutively admitted from May 2015 to December 2017; 52 patients were lost to follow-up, 311 patients were followed up for 2–3 years. The patients were aged from 65 to 99 years, with an average of 80.2 ± 6.5 years. There were 80 men and 231 women. There were 223 (72.9%) patients with internal diseases and 69 (22.5%) with three or more types of internal disease. The Charlson comorbidity index (CCI) was 0–6. The mean time from admission to surgery was 66.5 h, and 49.8% of patients had surgery within 48 h on admission. The average time from fracture to surgery was 4.3 days, 22.7% of patients had surgery within 48 h after fracture. One hundred and thirty-nine patients accepted Zoledronic Acid 5 mg (Aclasta) on their own free will less than 3 days after surgery.

The general data of living and dead patients were compared. The results showed that most of the dead patients were older, lighter weight, with more complications. However, the previous history of fragile fracture, total hip BMD, HGB, 25OHD had no difference between the dead and the living patients. The data are shown in Table 1. The average age of men was 80.7 ± 6.6 years old and that of women was 80.1 ± 6.4 years old. There was no significant difference in age between genders (*P* = 0.470). There were 4 males (5.0%) and 14 females (6.1%) over 90 years old.

**Table 1:** General condition of the femoral intertrochanteric fracture patients

	Case M/F	Age	Pre-fracture M/F	Re-fracture M/F	BMI g/cm^2^	Hip-BMD g/cm^2^	HGB g/l	25OHD ng/ml	CCI ≥ 3 n (%)	Zoledronic acid n (%)
Alive	274 (67/207)	79.8 ± 6.4	13/55	5/19	23.5 ± 3.6	0.666 ± 0.125	113.7 ± 17.0	12.5 ± 8.2	58 (21.2)	128 (46.7)
Death	37 (13/24)	83.4 ± 6.0	3/2	0/1	22.2 ± 4.0	0.647 ± 0.162	108.2 ± 20.5	11.0 ± 7.3	12 (32.4)	11 (29.7)
Total	311 (80/231)	80.2 ± 6.5	16/57	5/20	23.4 ± 3.6	0.664 ± 0.130	113.0 ± 17.4	12.3 ± 8.1	70 (22.5)	139 (44.7)
*P* value^a^	0.253	0.001	0.126	0.759	0.042	0.448	0.087	0.333	0.035	0.140

M, Male; F, female; BMI, body mass index; BMD, bone mineral density; HGB, hemoglobin; CCI, Charlson comorbidity index

^a^The compare between alive and death;

### All-cause mortality of cumulative survival rate

A total of 311 patients with fractures followed with an average time of 23.5 ± 5.0 months; 37 patients died during the follow-up period, the cumulative mortality after fracture was 10.8%, annual mortality for fracture was 5.4%. There were 13 men and 24 women among these 37 deaths, and the annual mortality was 6.1% in the male patients and 4.1% in the female patients. Of 37 deaths, 4 occurred within the hospital, 6 occurred within 3 months, 4 within 6 months, and 14 within 1 year after the fracture. The most common causes of mortality were cardiovascular events and pneumonia.

**Fig. 1:**
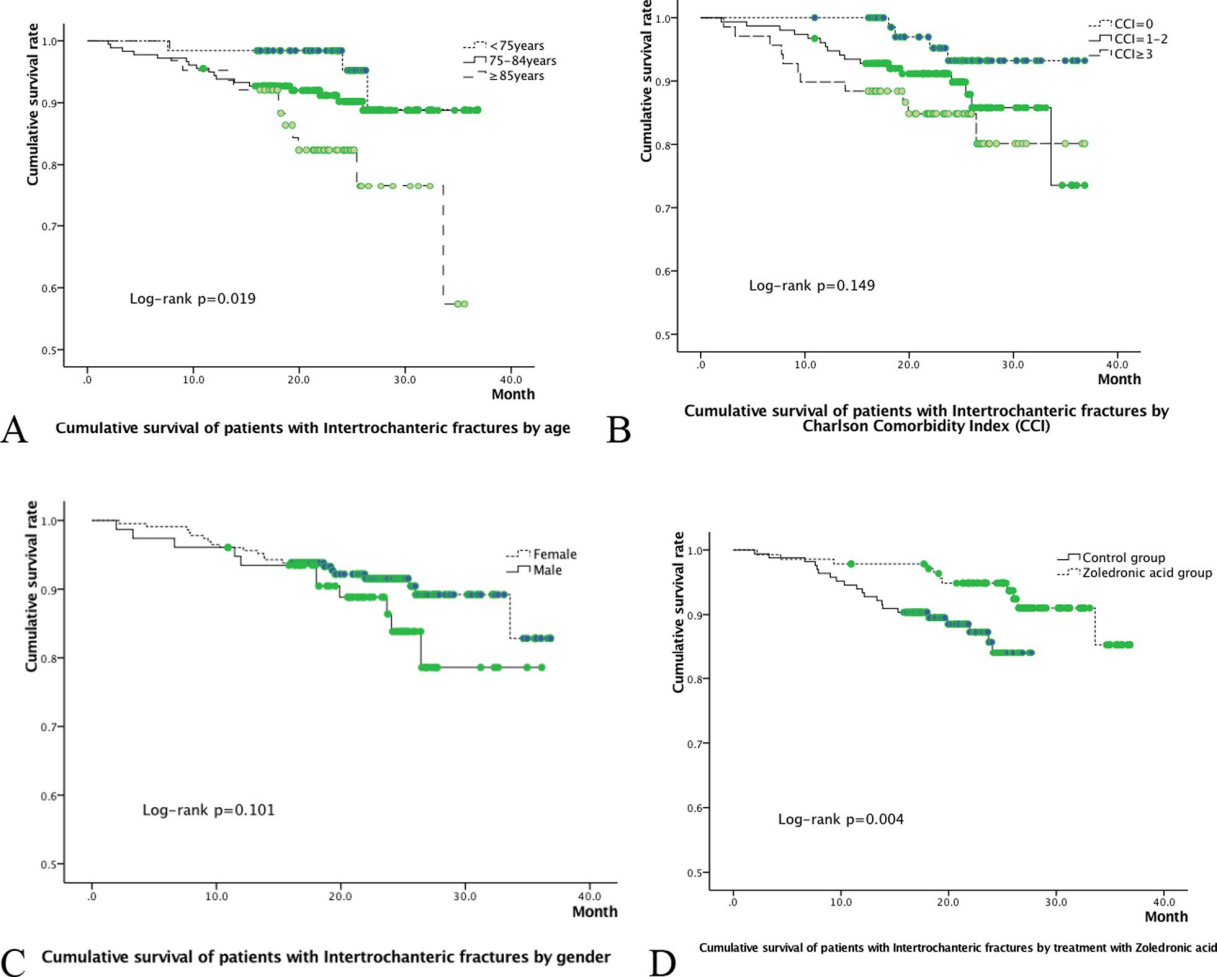
Cumulative survival of Intertrochanteric fracture patients by gender (**A**), age (**B**), Charlson comorbidity index (**C**) and dosing zoledronic acid or not (**D**)

Kaplan–Meier survival curves analysis showed that all-cause mortality increased in more elderly patients (*P* = 0.019, Fig. 1A); but only had a growing trend with CCI 3 or bigger (*P* = 0.149, Fig. 1B), and showed no gender difference (*P* = 0.101, Fig. 1C), and there was a correlation between using ZOL and the cumulative survival rate (*P* = 0.004, Fig. 1D).

### Multivariate cox regression analysis

The multivariate Cox proportional hazards model was applied to estimate the effects of factors on cumulative survival rate. The factors evaluated in this model included age, gender, BMI, fracture history, hip BMD, hemoglobin, 25OHD, CCI, and dosing ZOL. In this study, BMI, fracture history, hip BMD, hemoglobin, and 25OHD were not associated with all-cause mortality.

In Intertrochanteric fracture patients, during a mean observation period of 23.5 months, age (*P* = 0.022), CCI (*P* = 0.046), and early use of ZOL (*P* = 0.005) were associated with all-cause mortality; gender (*P* = 0.126) was not relevant (Table 2). In Intertrochanteric fracture patients, the elderly over 85 years had a 1.87-fold higher risk than the younger elderly, and those with a CCI index over 3 had a 1.63-fold higher risk of all-cause mortality than those with less than 3. In Intertrochanteric fracture patients, the early postoperative use of ZOL reduced the risk of all-cause mortality by 67%.

**Table 2:** Multivariate cox proportional analysis of risk factors for mortality in hip fracture patients

Type	Variable	HR (95% CI)	*P* value
Intertrochanteric fracture	Gender, male/female	–	0.126
	Age, ≥ 85/75 to 85/< 75 years	1.87 (1.10–3.19)	0.022
	CCI, ≥ 3/1 to 2/0	1.63 (1.01–2.63)	0.046
	ZOL, use/none	0.33 (0.16–0.71)	0.005

Blank cells indicate variables were not significant in the multivariate Cox proportional hazards models

CI, Confidence interval; HR, hazard ration; CCI, Charlson comorbidity index; ZOL, zoledronic acid

## Discussion

Most studies have treated hip fracture as a single, homogeneous condition, while hip fracture includes two major anatomic types: fractures of the intertrochanteric region and fractures of the femoral neck; the former is an extracapsular fracture, and the latter belongs to intracapsular fracture. And the trochanteric region has a greater proportion of trabecular bone than the femoral neck (50% vs 25%) [17]. The composition of bone in the two regions differs, so the etiology of each fracture type may also differ. A prospective study showed femoral neck and intertrochanteric fractures have different risk factors [18], BMD and poor functional ability largely predicted femoral neck fracture, while aging and poor health status predisposes to intertrochanteric fracture. Another prospective study showed that differences in patient characteristics and sequelae do exist between the femoral neck and intertrochanteric hip fracture patients that impact upon recovery. And it appears that intertrochanteric fracture patients have intrinsic factors (older age, poor health) impacting upon their risk of fracture and ability to recover [19]. A retrospective study showed mortality rate at 90 days was 12.1% for intertrochanteric fractures and 9.6% for femoral neck fractures [20]. Another prospective study even suggested that fracture type is an independent predictor of mortality in hip fracture patients, both at 1 month and at 1 year after injury [21]. So we should treat the intertrochanteric fracture and femoral neck fracture differently, and analysis their mortality rate separately at least. We discuss all-cause mortality risk in the aged femoral intertrochanteric fracture in this article.

In recent years, many guidelines and expert consensus recommend that the multi-department cooperative treatment group should be established in the treatment of hip fracture in elderly patients to improve perioperative safety, and operate as soon as possible (within 48 h), and then accelerate rehabilitation under the guidance of rehabilitation doctors [22–24]. In the process of multi-department collaborative treatment, the cooperation between orthopedics and geriatrics is important. Grigoryan et al. [25] summarized 18 studies through meta-analysis and found that the cooperative treatment of elderly hip fractures by orthopedics doctors and geriatricians can shorten the length of hospital stay and reduce in-hospital mortality and long-term mortality. So it is a more efficient way to establish a special ward and adopt the co-management model between orthopedics and geriatrics (or internal medicine) [26, 27]. With the co-management of orthopedics and geriatrics, an all-cause mortality rate of patients included in this article decreased significantly, the 1-year mortality rate was 4.5% compared with 17.47%, which was from a systematic analysis data after femoral intertrochanteric fracture between the years 2000 and 2018 in Mainland China [1]. Therefore, this article explored the risk factors of all-cause mortality under this model.

Under the co-management model of orthopedics and geriatrics, from our data, risk factors of all-cause mortality were older age, CCI, and early use of ZOL; they were consistent with previous studies [28–31]. While male gender was not a risk factor. It is speculated that different data have different gender and age distribution; it is also possible that the number of data in this paper is still insufficient and cannot show the difference. Old age is an important predictor of all-cause mortality, even accounting for 3–4 points in NHFS score [16]. Charlson proposed CCI in 1987, 19 concomitant diseases that significantly affect the survival time are weighted according to the severity, which can well reflect the comorbid state of patients.

To the dosing of ZOL, we summarized different studies about all-cause mortality after bisphosphonates treatment in patients with hip fracture (Table 3), from the hazard ratios, it seems China's hip fracture population had better effects. The influence of ethnicity is huge. Such as, Chinese people are prone to atypical fracture after continuous BPs [32–34]. It may also be related to the better effect in patients with femoral intertrochanteric fracture.

**Table 3:** Simple summary of different studies on improving all-cause mortality after bisphosphonates treatment in patients with hip fractures

References	Anti-OP drug	Use time	Frequency	Fracture type	Race or region	Research type	Treated cases N	Age	Mean follow-up time (M)	HR (95% CI)	*P* value
Lyles et al. [45]	ZOL	≤ 90 days after surgical	Yearly	Hip fracture	No Asia	Randomized, double-blind, placebo-controlled trial	1065	≥ 50	22.8	0.72 (0.56, 0.93)	0.01
Eriksen et al. [46]	ZOL	≤ 2 week after surgical	Yearly	Hip fracture	No Asia	Randomized, double-blind, placebo-controlled trial	56	≥ 50	22.8	–	> 0.05
Bergman et al. [47]	ZOL	–	–	Hip fracture	Sweden	Retrospective cohort study	161	≥ 50	33.6	1.51 (1.00, 2.28)	0.048
Wang et al. [48]	ZOL	≤ 2 weeks after admission	–	Hip fracture	China	Retrospective study	80	≥ 50	28.5	0.36 (0.19, 0.65)	< 0.01
Brozek et al. [49]	All BP and Denosumab	≤ 1 year	–	Hip fracture	Austria	Retrospective nationwide cohort study	2166	≥ 50	36	0.48 (0.42, 0.55)	< 0.0001
Bergman et al. [47]	Alendronate	–	–	Hip fracture	Sweden	Retrospective cohort study	4689	≥ 50	33.6	0.82 (0.76, 0.89)	< 0.001
Bondo et al. [50]	Oral BP	5 month after fracture	> 2 packs	Hip fracture	Danish	Nationwide register-based open cohort study	1096	≥ 55	45.6	0.73 (0.61, 0.88)	0.001
Axelsson et al. [51]	Alendronate	–	≥ 3 months	Hip fracture	Sweden	Prospective observational register-based study	1961	≥ 80	19.2	0.88 (0.82, 0.95)	< 0.01
Beaupre et al. [52]	Oral BP	1 and 2 years	64% yearly	Hip fracture	Canada	Randomized controlled trial	101	> 50	36	0.92 (0.88, 0.97)	0.001
Van Geel et al. [53]	Oral BP	2 weeks later	Continued 5 years	Clinical fracture (21.7% for hip fracture)	Scotland	Prospective cohort study	2534	≥ 50	44.9	0.79 (0.64, 0.97)	0.021
This study	ZOL	≤ 3 days after surgical	Once	Intertrochanteric fracture	China	Prospective cohort study	139	≥ 65	23.5	0.33 (0.16–0.71)	0.005

– Indicate variables were not significant in the multivariate Cox proportional hazards models

OP, Osteoporosis; HR, hazard ration; CI, confidence interval; ZOL, Zoledronic acid; BP, Bisphosphonates

As we know, bisphosphonates (BPs) bind to hydroxyapatite crystals in bone, especially at sites with high bone turnover [35], which means BPs bind strongly at sites of new mineral deposition, also binds well to resorption sites. And more BPs is taken up by trabecular bone than cortical bone, for a higher rate of turnover and greater surface area available in trabecular bone [36]. BPs released from bone may undergo re-uptake onto bone surfaces, so they can be detected in urine for years after treatment discontinuation [37, 38]. Patents healing after fracture is in the period of rich callus formation, and their bone metabolism is more active; therefore, after using ZOL during fracture healing, patients will retain more ZOL in the bone, which can play a more effective and longer anti-osteoporosis effect. As there is more bone trabecular in the intertrochanteric region, and bone turnover markers are higher after operation of intertrochanteric fracture [39], we have reasons to believe that ZOL deposit more in intertrochanteric fracture, so it may also affect the long-term prognosis.

However, we cannot consider that ZOL reduces the all-cause mortality of patients with femoral intertrochanteric fracture by reducing secondary fractures. A meta-analysis collected all randomized controlled trial study of osteoporosis agents with proven anti-fracture indicated that mortality risk reduction was not associated with the reduction in the incidence of a new hip, vertebral or non-hip non-vertebral fracture [40]. And, there are clinical, animal, and molecular studies that proved immune-modulatory effects; bone loss and bone turnover decreasing; fibrosis and apoptosis effects are all had effects on decreasing all-cause mortality [41].

The feature of this study was that ZOL was used within 3 days after surgery, which is early; most of the published studies on the use of BPs after hip fracture have been used for 2–4 weeks or later. On the other hand, it verified that the early use of ZOL did not bring more adverse. Another feature of the study was that ZOL was used only once during the observation period of 2–3 years, due to the less attention paid to osteoporosis treatment by patients and their families and the inconvenient activities of patients. For ZOL effects on BMD and fracture risk persisted for at least 2 years [42–44], which makes ZOL a more attractive proposition. This design also avoids immortal time bias, multiple medication bias, the arbitrary time point of administration, and adherence bias.

## Conclusion

Determination of risk factors supports doctors to identify patients who were at high risk for mortality and enables accurate preoperative risk assessment. We should pay attention to the patients with femoral intertrochanteric fracture over 85 years old and CCI greater than or equal to 3, and give ZOL and basic anti-osteoporosis treatment in time.

## Limitations

This study has two major limitations. First, this is a single-center study, the sample size was relatively small, while data are homogeneous thus eliminating the potential confounding factors; however, a multi-centric study with more patients evaluated could better address the risk factor in elderly patients who have undergone a hip fracture. Second, some data are incompletely reported, and this could influence the evaluation of data.

## Data Availability

Not applicable.
